# Protocol for a systematic review of prognostic models for recurrent events in chronic conditions

**DOI:** 10.1186/s41512-020-0070-9

**Published:** 2020-01-28

**Authors:** Victoria Watson, Catrin Tudur Smith, Laura Bonnett

**Affiliations:** Department of Biostatistics, University of Liverpool, Liverpool, UK

**Keywords:** Prognostic factors, Prognostic models, Recurrence, Chronic condition, Prediction, Validation, Meta-analysis

## Abstract

**Background:**

Prognostic models for repeated events of the same type are highly useful in predicting when a patient may have a recurrence of a chronic disease or illness. Whilst methods are currently available for analysing recurrent event data in prognostic models, to our knowledge, most are not widely known or applied in a medical setting. As a result, often only the first recurrence is analysed meaning valuable information for multiple recurrences is discarded. Therefore, the aim of this review is to systemically review models for repeated medical events of the same type, to determine what modelling techniques are available and how they are applied.

**Methods:**

MEDLINE will be used as the primary method to search sources. Various databases from the Cochrane Library and EMBASE will also be searched. Trial registries such as Clinicaltrials.gov.uk will be searched, as will registered trials that are ongoing and not yet published. Abstracts submitted to conferences will also be searched, and non-English sources will also be considered. Studies to be included in the review will be decided based on PICO guidelines, where the study population and outcomes correspond to this study’s aims and target population. The prognostic models used in each study chosen for inclusion in the review will be summarised qualitatively.

**Discussion:**

As recurrent event data is not widely analysed in prognostic models, the results from this systematic review will identify which methods are available and which are commonly used. It is also unknown if certain methods which will be identified in the review perform better given certain conditions. Therefore, if included studies assess predictive performance, the results of this review could also provide evidence to determine if certain models are better fitting dependant on the event rate of the chronic condition. The results will be used to determine if model selection varies across disease area. The review will also provide an insight into the development of any new methods used for analysing recurrent events.

**Trial registration:**

The review has been registered on PROSPERO (CRD42019116031).

## Background

A chronic condition is defined as a long-term condition affecting physical health. Although medication and/or therapies can be taken to control the condition, there is no cure for a chronic disease or condition [1]. It is estimated that 17.5 billion adults in the UK live with a chronic condition such as epilepsy, asthma and cardiovascular diseases [2]. Many people who live with a chronic disease or condition are at risk of multiple recurrences throughout their life time. Being able to predict when this recurrence may occur would be highly beneficial to clinicians and patients in terms of treatment choice and patient counselling.

A prognostic factor is a factor which is used to predict the risk of a recurrence of a chronic disease or condition, or the chance of recovery [3]. Multiple prognostic factors can be combined in prognostic models. These predict outcome for patients, calculating the probability the patient has of developing a disease or recurrence in the future [4]. The results can be utilised to determine patients who are at a high risk of a recurrence and thus determine if a patient requires alternative or additional treatment or intervention. Likewise, the results from the models can also be used to identify patients who are at low risk of a recurrence and therefore may require less frequent follow-up. Currently, prognostic models for recurrent event data are not widely utilised or developed in research, and commonly only the time until the first event or recurrence is analysed [5]. However, as patients living with chronic conditions have numerous recurrences over a lifetime, only being able to predict the time until the first recurrence is not particularly informative, and a lot of key clinical information is lost. Performing a systematic review of prognostic models for recurrent data will identify and summarise models available and applied in research.

Prognostic factors can be useful in identifying groups of patients who are at highest risk of recurrence but can also be combined in prognostic models to predict individual level of risk of a chronic condition or disease at a certain timepoint assuming baseline covariates. Therefore, the results of our systematic review will evaluate existing methodology and determine whether the identified methods differ dependant on the event rate of the chronic condition. This information can then be utilised to provide evidence for future use of such methods, informing researchers which methodology may be best suited to the chronic condition they are modelling.

## Methods/design

### Research aims

The aim of this systematic review is to identify methods used in prognostic modelling of recurrent event data. This will be achieved by summarising qualitatively which models were used and how they were applied. If data is found to be available, model performance and predictive capability of disease recurrence and other outcomes of each of the prognostic models applied will also be summarised.

### Study design

Studies which use and/or develop prognostic models for recurrent event data to predict the risk of a recurrence will be assessed for inclusion in the review. No limit will be placed on the specific type of study design, as randomised controlled trials, cohort studies and case control studies will all be assessed for inclusion in the review.

### Study population

The study population will not be defined by a certain age group or ethnicity. However, the study population must include patients who are at risk of experiencing recurrent events in chronic conditions.

### Study outcomes

Studies with any outcome regarding recurrent events will be included, specifically recurrence of symptoms which typify a chronic condition such as seizures in epilepsy or attacks in asthma.

### Setting

No country-specific databases will be searched, as there is no set study population geographically. However, the country which the database represents will be considered when evaluating studies, as will whether the study was conducted in higher-, lower- or middle-income countries. This is to account for potential differences in event rates due to possible varying standards of care across countries. Access which the studied population may have to treatment may also be an important factor regarding event rate, as those in lower income countries may not have treatment or therapy widely available to them and therefore have a higher event rate.

### Study selection

This is a methodological review and the specific disease area will not be specified when deciding which articles will be included, providing the study is evaluating a chronic condition or disease where the risk of recurrence is the primary area of interest. Studies will be compared against a developed eligibility checklist to determine if they will be included in the systematic review. A standardised search filter will be used when searching studies as part of the literature review—more details of this can be found below in the “Search approach” section—and any full texts which are referenced in papers will also be evaluated. Key authors of papers will be contacted for further information if necessary [6].

### Primary and secondary outcomes

The primary outcome of this systematic review is to identify methods used within prognostic modelling for recurrent event data. A secondary outcome, if reported upon in included studies, is to summarise model performance in terms of how accurate the model predicts recurrence of a chronic condition or disease. Examples of these performance measures include discriminatory statistics such as the C-Statistic or Brier score and calibration statistics such as the calibration slope. Secondary outcomes will also evaluate the predictive capability of the prognostic models used to predict not just the recurrence but other relevant outcomes. These results will not only inform future researchers what models there are available for analysing recurrent event data in prognostic models but will also provide evidence regarding what model may be better suited to their data.

### Search approach

Bibliographic databases will be searched for studies to include in the review, with the primary source of interest being the Medical Literature Analysis and Retrieval System Online (MEDLINE). The Cochrane Library (Wiley) which will include the Cochrane Database of Systematic Reviews (CDSR), Health Technology Assessment (HTA) Databases, and Database of Abstracts of Review of Effects (DARE) as well as Cochrane Controlled Register of Trials (CENTRAL) will also be searched to identify any potentially similar systematic reviews from which further appropriate sources can be identified from. The Excerpta Medica Database (EMBASE) will also be searched. Searches will be performed using index terms and phrases related to recurrent events and prognostic factors. The full search strategy can be seen in the Appendix.

Public trial registers will also be searched such as Clinicaltrials.gov for registered trials in the UK as will the World Health Organization (WHO) International Clinical Trial Registry to search trials outside of the UK. Other trial registries will include, but are not limited to, UK Clinical Research Network Study Portfolio Database (UKCRN) and *meta*Register of Controlled Trials (mRCT). This will also enable ongoing trials that are not yet published to be searched [7].

References cited in identified sources will be examined, and the full papers will be searched to determine if the full text is available. If necessary, the key authors of the paper will be contacted. Sources of all languages and time periods will be searched. To identify other studies which have not been published yet, abstracts for conferences relevant to our research will be searched which apply prognostic models for recurrent events. Systematic reviews found which are relevant will also be searched for further studies to include [6]

### Study selection

The initial screening procedure will consist of two independent reviewers (VW and LJB) who will screen titles and abstracts, removing any which they feel are not relevant. This will be done using pre-defined screening criteria. A key part of these criteria will be if prognostic modelling methods are used for recurrent event data. Additionally, the endpoint of the study must be outcomes associated with recurrences of symptoms which typify a chronic disease or condition, for example angina attacks in heart disease. Studies chosen for inclusion may model the number of recurrent events or the time between recurrent events; other studies related to multiple events will also be considered. Once titles have been screened, the full texts will be obtained from those selected and reviewed by the two independent reviewers separately against full eligibility criteria. In the case of non-English reviews being considered, relevant sections will be translated. Discrepancies between reviewers that cannot be resolved by discussion will be discussed with a third reviewer (CTS). The reviewers’ decisions and reasons for exclusion will be recorded using appropriate reference management software such as EndNote [8]. The review process will be documented using the Preferred-Reporting Items for Systematic Reviews and Meta-Analyses (PRISMA) flow diagram [9].

### Data extraction

A detailed data extraction form will be developed prior to the systematic review being performed. The data extraction will be checked by a second reviewer (LJB) by randomly selecting 10% of studies. Discrepancies will be clarified by a third reviewer (CTS). The Checklist for critical Appraisal and data extraction for systematic Reviews of prediction Modelling Studies (CHARMS) will be utilised at this stage. Study characteristics such as sample size, country and year will be extracted onto this form, as will study design characteristics which will include the medical condition under consideration, the design of the study and length of follow-up. The event rate will also be recorded, for example how many events were observed within the study period and the annual event rate if possible. Whether the design of the study has any risk of bias will also be recorded. Primary and secondary outcomes of the study will be recorded, for example recurrence of disease along with the disease area or chronic condition being investigated.

Prognostic factors included in the model will be recorded and how they were measured and incorporated into the model. Whether the prognostic factors in the model were continuous, categorical, binary or count data and whether continuous variables have been dichotomised with a description of how they were derived where possible will be recorded. At which point in time when a patient is defined as being at risk again after the first or previous recurrence will be extracted, allowing for the time measurement of the prognostic factors to be captured. The prognostic model used in the final analysis and justifications for how the model was chosen for analysis will be recorded. Any reported strengths and weaknesses of the applied method will be extracted, and if multiple methods are applied in the same study which method was concluded to be the best performing with justifications will also be extracted if provided. Similarly, details regarding how the model was applied will be extracted. How the model expresses individual outcome risk will also be extracted such as the hazard ratio (HR) or incidence rate ratios (IRR) along with the associated confidence intervals, which will also allow for the magnitude of the effect and degree of uncertainty to be summarised. If it is found that studies apply multiple recurrent event methods to the same dataset and report multiple results, extracting measures regarding the magnitude of the effect size and degree of uncertainty will allow us to determine if a certain method over- or underestimated the effect when compared to others or had a higher degree of uncertainty for example. When pooling the results together, narratively, it could be that this was a common occurrence for a method identified.

Whether the study analysed the first recurrence in standard Cox regression for example as a comparator to recurrent event analysis will be extracted, as will the reported effect estimate and confidence interval. This will allow for a comparison to the recurrent event analysis methods by assessing the magnitude and direction of the reported estimated effect size and degree of uncertainty.

Any internal or external model validation to assess model performance will also be described if reported, such as if any discrimination statistics such as the C-Statistic or area under the curve (AUC) were used to assess how well the model distinguishes between those who had the event to those who never did and, similarly, if any calibration statistics were reported such as the observed/expected event ratio to assess the level of agreement between the two [7]. This can be used to assess how well the identified methods for recurrent event analysis perform and could also be used to compare model and predictive performance of other recurrent event models.

### Assessment of study quality

Publication bias, selection bias and language bias will all be taken into consideration when reviewing studies. Risk of bias will be evaluated using guidelines proposed by Altman [10], and the risk Prediction model Risk Of Bias ASsessment Tool (PROBAST) [11] and the QUality in Prognosis Studies (QUIPS) tool will also be utilised [12].

Study design, sample size, analysis methods and missing data will be evaluated to determine the quality and reliability of each study. The reliability of the prognostic factors and study outcomes used in the models will be assessed. In terms of sample size, higher quality studies will be identified as those which use a pre-specified sample size considering the expected number of events when the sample size and power calculation was performed. The sample size calculation should also allow for multiple comparisons between factors.

Higher quality studies will be identified as those which have minimal loss to follow-up in the study meaning the majority of the data is available for model validation. Whether internal and external model validation was performed and whether a clear description of this is provided will be regarded of a higher quality than those that do not contain this. Transparency of the data quality should be evident as should any missing data and how it was addressed in the study, for example the use of imputation should be clearly specified and defined including the number of imputations used [7].

The quality of the analysis performed will be assessed based on how prognostic factors were chosen for the model and how they were measured in the data capture process. If overfitting of the model and model optimism was accounted for using bootstrapping and shrinkage will also be evaluated. Whether the prognostic factors were continuous or if they had been dichotomised will also be taken into consideration as dichotomised variables may not be as efficient as continuous predictors. Although the primary aim is not to assess the quality of included studies, there is a need to summarise the quality, as low-quality studies may not be using the most appropriate statistical method for example [13]. Similarly, any conclusions drawn from the included study regarding the results and performance measures of the identified recurrent event method should be interpreted with caution for lower-quality studies.

### Evidence synthesis

A narrative synthesis will be provided for studies included in the review. Appropriate data will be presented in the form of summary tables and where relevant graphical representations of the data will be provided.

## Discussion

The results of this systematic review will identify the statistical models available for prognostic modelling of recurrent event data and which of these are most commonly applied across different clinical areas. Recording data regarding the predictions from the models such as the reported risk of a recurrence and degree of uncertainty allows us to determine if certain methods identified in the review tended to commonly provide biased estimates compared to others for example. This will be applicable to studies which apply multiple recurrent event methods to the same dataset allowing us to compare between them. Extracting any performance measures which may be reported will also provide insight into the predictive capability of the methods identified. The reported strengths and weaknesses will be recorded as will the event rate of the conditions being modelled. This will allow us to determine if certain models work well dependant on their event rate in the observed study period. A review of how each of these models is applied will also be provided, to distinguish any similarities or differences in reporting between the models and across clinical areas.

Not focussing on a specific disease area or chronic condition allows us to examine any potential trends or patterns within clinical areas to determine if a specific prognostic model tends to be more commonly applied when modelling certain diseases or chronic conditions, thus allowing for a comparison across clinical areas to be made. As a result, models for recurrent event data which are not widely applied in research will also be identified, thus identifying gaps for potential future research into the statistical modelling applications.

Therefore, this review will provide evidence of prognostic models available for recurrent event data, allowing for the optimisation of analysis of recurrent event data in the future.

## Data Availability

Not applicable

## Appendix

**Table 1:** Search strategy for MEDLINE

No.	Search term	Field
1	Predict*	All fields
2	Model*	All fields
3	Statistical	All fields
4	Prognos*	All fields
5	Risk	All fields
6	Analy$ing	All fields
7	Survival	All fields
8	OR 1-7	All fields
9	Repeat*	All fields
10	Recur*	All fields
11	Multiple adj3 Failure	All fields
12	“Multiple time-to-event”	All fields
13	OR 9-12	All fields
14	“Extended Cox”	All fields
15	Andersen Gill	All fields
16	Anderson Gill	All fields
17	Prentice Williams Peterson	All fields
18	Wei Lin Weissfeld	All fields
19	Shared Frailty	All fields
20	“Joint Frailty”	All fields
21	“Proportional Intensity model”	All fields
22	“Multi State”	All fields
23	OR 14-22	All fields
24	8 AND (13 OR 23)	All fields

## Abbreviations

AUC: Area under the curve
CDSR: Cochrane Database of Systematic Reviews
CENTRAL: Cochrane Controlled Register of Trials
CHARMS: CHecklist for critical Appraisal and data extraction for systematic Reviews of prediction Modelling Studies
DARE: Database of Abstracts of Review of Effects
EMBASE: Excerpta Medica Database
HR: Hazard ratio
HTA: Health technology assessment
IRR: Incidence rate ratio
MEDLINE: Medical Literature Analysis and Retrieval System Online
mRCT: *meta*Register of Controlled Trials
PRISMA: Preferred-Reporting Items for Systematic Reviews and Meta-Analyses
PROBAST: Prediction model Risk Of Bias ASsessment Tool
PROSPERO: International Prospective Register of Systematic Reviews
QUIPS: QUality in Prognosis Studies
UKCRN: UK Clinical Research Network Study Portfolio Database
WHO: World Health Organization
